# TRPM4 inhibition slows neuritogenesis progression of cortical neurons

**DOI:** 10.1186/s13041-024-01140-3

**Published:** 2024-09-12

**Authors:** Denise Riquelme, Nicole Juanchuto-Viertel, Carlos Álamos, Elias Leiva-Salcedo

**Affiliations:** Department of Biology, Faculty of Chemistry and Biology, University of Santiago of Chile, Santiago, Chile

**Keywords:** TRPM4, Neuritogenesis, Cortical neuron development, Intracellular calcium

## Abstract

TRPM4 is a non-selective cation channel activated by intracellular Ca^2+^ but only permeable to monovalent cations, its activation regulates membrane potential and intracellular calcium. This channel participates in the migration and adhesion of non-excitable cells and forms an integral part of the focal adhesion complex. In neurons, TRPM4 expression starts before birth and its function at this stage is not clear, but it may function in processes such as neurite development. Here we investigate the role of TRPM4 in neuritogenesis. We found that neurons at DIV 0 express TRPM4, the inhibition of TRPM4 using 9-Ph reduces neurite number and slows the progression of neurite development, keeping neurons in stage 1. The genetic suppression of TRPM4 using an shRNA at later stages (DIV2) reduces neurite length. Conversely, at DIV 0, TRPM4 inhibition augments the Cch-induced Ca^2 +^ _i_ increase, altering the calcium homeostasis. Together, these results show that TRPM4 participates in progression of neurite development and suggest a critical role of the calcium modulation during this stage of neuronal development.

**Supplementary Information:**

The online version contains supplementary material available at 10.1186/s13041-024-01140-3.

## Introduction

Neuritogenesis is the process by which neurons undergo morphological changes leading to neurite formation, allowing them to acquire a polarized morphology and establish somatic, dendritic, and axonal compartments [1]. This is the first step in neuronal development, particularly in excitatory cortical neurons, occurring alongside their migration from the ventricular zone to cortical areas [2].

Neurite formation begins with F-actin rearrangement, leading to membrane protrusion and lamellipodia and filopodia formation. During engorgement, microtubules advance into the lamellipodia and filopodia, leading to cylindrical neurite consolidation [3]. This process is highly regulated by intracellular Ca^2+^ (Ca^2 +^ _i_) [4, 5]; its absence arrests filopodia formation and neurite progression [6]. Inhibitors of Ca^2 +^ _i_ release or inhibitors of voltage gated calcium channels reduce neurite outgrowth [7], and complete suppression of Ca^2 +^ _i_ causes growth cone loss and cessation of neuritogenesis in cerebellar neurons [8]. Thus, ion channels and transporters affecting the resting membrane potential and intracellular calcium also regulate neurite elongation and neuronal development [9].

Calcium signals and membrane potential regulation are critical for neuritogenesis; changes in both can reduce migration and alter neurite number and development. TRPM4, a non-selective cation channel activated by Ca^2 +^ _i_ and permeable to monovalent cations, modulates calcium levels by controlling the Ca^2+^ driving force [10], or indirectly by activating voltage-gated calcium channels through depolarization [11]. TRPM4 regulates focal adhesion by modulating Ca^2 +^ _i_ in non-excitable cells, localizing in focal adhesions, with its trafficking regulated by microtubule-tracking protein EB, participating in focal adhesion disassembly and migration [12, 13]. Studies indicate TRPM4 is critical for adhesion and migration of various non-neuronal cells, such as mastocytes, lymphocytes, and melanoma cell lines. TRPM4 is expressed in the prefrontal cortex at least from P0 [14], suggesting a potential role in development. However, its specific role in neuritogenesis has not been explored. This study assesses TRPM4 role in neuritogenesis and Ca^2 +^ _i_ dynamics during early neurite development.

## Results

**Fig. 1:**
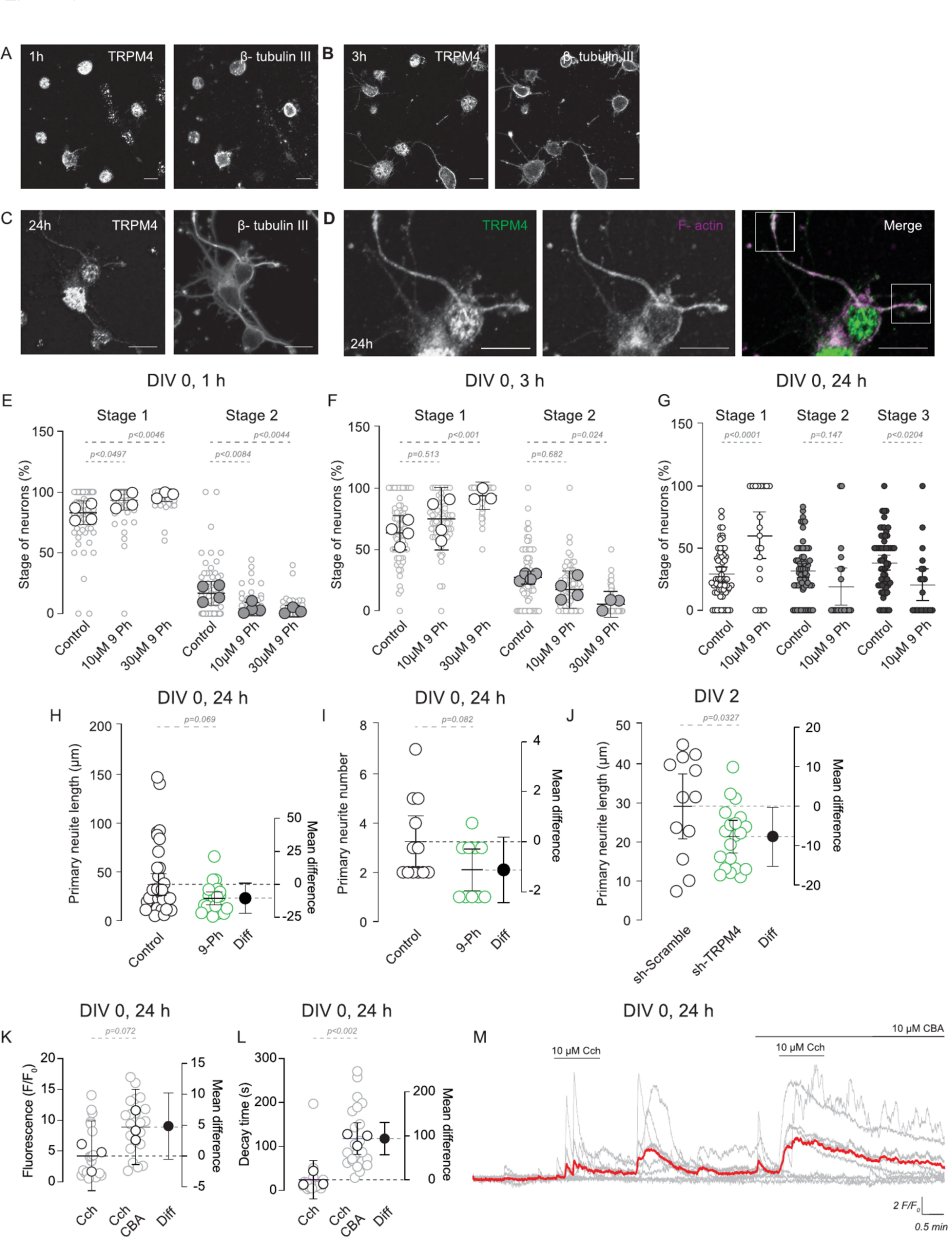
TRPM4 inhibition reduces neurite development progression of cortical neurons. In (**A**) Immunostaining with TRPM4 and β-tubulin III at 1 h and (**B**) 3 h and (**C**) 24 h post disaggregation. (**D**) Zoom of the (**C**) showing the Immunostaining of TRPM4 and F-actin at 24 h, the white box indicates the expression in the growth cone. Scale bar = 10 μm. (**E**) Summary graph showing the effect of TRPM4 inhibition with 10 µM 9-Ph and 30 µM 9-Ph in the percentage of neurons in stage 1 and 2 of the neuritogenesis after 1 h (*N* = 4). (**F**) Summary graph showing the effect of TRPM4 inhibition with 10 µM 9-Ph and 30 µM 9-Ph in the percentage of neurons in stage 1 and 2 of the neuritogenesis after 3 h (*N* = 4). (**G**) Summary graph showing the effect of TRPM4 inhibition with 10 µM 9-Ph in the percentage of neurons in stage 1, 2 and 3 of the neuritogenesis after 24 h post culture (*N* = 2). Summary graph showing the length (**H**) and number (**I**) of the primary neurite before and after 10 µM 9-Ph. (**J**) Shows the primary neurite length in neurons transfected with shRNA-TRPM4 and the scramble sequence. Summary graph showing the quantification of the (**K**) maximum fluorescence and the (**L**) decay time of the signal induced by 10 µM Cch and 10 µM Cch + 10 µM CBA (*N* = 3). (**M**) Representative graph showing the Ca^2 +^ _i_ response, gray lines showing all neurons recorded, red line shows the average of the signal. Data is shown as the mean ± 95 C.I. and the right plot shows the mean difference, the *p*-value is shown above the graph

To determine TRPM4 role in neuritogenesis, we assessed its expression in neurons during early development in culture. We performed immunostaining for TRPM4 and β-tubulin III in cortical neuron cultures at 1, 3, and 24 h after plating. TRPM4 was already expressed in neurons at 1 h after plating, with homogeneous expression in the soma. This pattern changed to somatic and neuritic localization at 3 h and became more pronounced at 24 h (Fig. 1A, B, and C).

To determine whether TRPM4 is coexpressed with F-actin at focal adhesions, a critical site for neurite outgrowth, we immunostained TRPM4 and used Phalloidin to detect F-actin. TRPM4 was coexpressed with F-actin, particularly in neurite growth cones (Fig. 1D), suggesting a role in neuritogenesis.

To examine the effect of TRPM4 inhibition on neuritogenesis, we used the model of polarity establishment with five stages: Stage 1, where spherical neurons start to extend filopodia and lamellipodia; Stage 2, where neurons present minor neurites; Stage 3, where one neurite grows to become an axon; and Stages 4 and 5, where neurons develop dendrites and form synapses, respectively [15]. We inhibited TRPM4 with 9-Phenanthrol (10 and 30 µM) in cortical neurons right after plating. After 1, 3, or 24 h of treatment, we fixed the neurons, immunolabeled with β-tubulin III and Phalloidin, then imaged neuronal morphology using a confocal microscope, and classified the neurons in the neuritogenesis stages (Suppl. Methods).

We found that 1-hour 9-Ph treatment slows the progression to Stage 2 (Control = 16.9%, 10 µM 9-Ph = 4.1%, 30 µM 9-Ph = 2.1%, Fig. 1E) and increases the percentage of cells in Stage 1 (Control = 83.1%, 10 µM 9-Ph = 93.4%, 30 µM 9-Ph = 97.8%, Fig. 1E). After 3 h of 9-Ph treatment, the percentage of neurons in Stage 2 is still reduced (Control = 27.7%, 10 µM 9-Ph = 17.9%, 30 µM 9-Ph = 5.7%, Fig. 1F), but there is an increase in neurons in Stage 1 (Control = 64.1%, 10 µM 9-Ph = 75.3%, 30 µM 9-Ph = 94.3%, Fig. 1F). After 24 h of treatment, we found that TRPM4 inhibition with 9-Ph slows neuritogenesis (Stage 1 Control = 31.2%, 10 µM 9-Ph = 60.1%, Fig. 1G), however, when we measure cell viability, we found that 24 h 9-Ph treatment induces cell death and decreases the number of the attached neurons (Suppl. Figure 1A). This suggest that the long-term treatment with 9-Ph affect neuronal adhesion and promote neuronal death.

Next, we treated neurons for 24 h with 10 µM 9-Ph and measured the primary neurites’ length and number. We found that 9-Ph decreases the length (Control = 37.3 μm, 9-Ph = 23.1 μm, Fig. 1H) and number (Control = 3.2, 9-Ph = 2.1, Fig. 1I) of primary neurites. To corroborate this, we transfected neurons with shRNA against TRPM4 and measured primary neurite length at DIV2, this procedure reduces TRPM4 expression in 54.3% (Suppl. Figure 1B). We found that TRPM4 silencing decreases neurite length (shScramble = 29.3 μm, shTRPM4 = 21.1 μm, Fig. 1J). We also tested the Ca^2 +^ _i_ response and found that TRPM4 inhibition with CBA increases decay time (Cch = 24.5 s, Cch + CBA = 118.2 s, Fig. 1L and M), thus increasing the total Ca^2 +^ _i_ mobilized.

## Discussion

TRPM4 is involved in non-neuronal cell migration, forming part of focal adhesions and regulating cell movement. Gradients of Ca^2 +^ _i_ are critical to promote neuritogenesis [6, 16], particularly low levels of Ca^2 +^ _i_ promote neurite outgrowth [6], in this context, our experiments shows that TRPM4 inhibition increases Ca^2 +^ _i_, thus may produce an opposite effect, and altering the optimal level of Ca^2 +^ _i_ necessary for neuritogenesis. This Ca^2 +^ _i_ increase may be due to the membrane potential of the neuron, which at this early stage is more depolarized and less responsive, since TRPM4 is expressed at least from DIV0 [14], TRPM4 could play critical roles in controlling depolarization and the Ca^2 +^ _i_ influx through VGCC during initial stages of development and impacting neurite outgrowth.

TRPM4 is expressed in lamellipodia where the presence of F-actin is critical for cell migration, our results shows that TRPM4 is coexpressed in areas rich in F-actin in the growth cone, thus in this area TRPM4 may participate in the tight control of the Ca^2 +^ _i_ which is critical for neurite outgrowth [6]. In non-excitable cells, TRPM4 participates in the focal adhesion turnover by modulating EB proteins, reducing focal adhesion disassembly, and decreasing lamellipodia extension [12, 13]; a similar effect may be playing TRPM4 in neurite outgrowth. We found that inhibiting TRPM4 with 9-Ph at the beginning of neuritogenesis is relevant for neurite stage progression. However, after 24 h inhibition, we observed no significant change in neurite length or number. Interestingly, TRPM4 silencing for 48 h significantly reduce neurite length, suggesting that TRPM4 also participates in later stage of neuritogenesis. In this context the use of strategies to silence the channel at earlier stages are necessary to complement our pharmacological approach and to fully investigate the effects of TRPM4 on the initial developmental stages of neuritogenesis. In conclusion, our findings suggest that TRPM4 plays a significant role in neuritogenesis by modulating intracellular calcium.

## Electronic supplementary material

Below is the link to the electronic supplementary material.

**Supplementary Material 1:** Supplementary Methods

**Supplementary Material 2:** Supplementary Figure 1

## Data Availability

All data generated or analyzed during this study are included in this published article.

## References

[1] Sakakibara A, Hatanaka Y. Neuronal polarization in the developing cerebral cortex. Front Neurosci [Internet]. 2015 [cited 2024 Jun 3];9. https://www.frontiersin.org/journals/neuroscience/articles/10.3389/fnins.2015.00116/full 10.3389/fnins.2015.00116 PMC4389351 25904841

[2] Kon E, Cossard A, Jossin Y. Neuronal Polarity in the Embryonic Mammalian Cerebral Cortex. Front Cell Neurosci [Internet]. 2017 [cited 2024 Jun 3];11. https://www.frontiersin.org/articles/10.3389/fncel.2017.00163 10.3389/fncel.2017.00163 PMC5472699 28670267

[3] Flynn KC. The cytoskeleton and neurite initiation. Bioarchitecture. 2013;3:86–109. 24002528 10.4161/bioa.26259 PMC4201609

[4] Bando Y, Irie K, Shimomura T, Umeshima H, Kushida Y, Kengaku M et al. Control of Spontaneous Ca ^2+^ Transients Is Critical for Neuronal Maturation in the Developing Neocortex. Cereb Cortex [Internet]. 2016 [cited 2021 Jul 2];26:106–17. https://academic.oup.com/cercor/article-lookup/doi/10.1093/cercor/bhu180 10.1093/cercor/bhu180 25112282

[5] Da Silva JS, Dotti CG. Breaking the neuronal sphere: regulation of the actin cytoskeleton in neuritogenesis. Nat Rev Neurosci [Internet]. 2002 [cited 2019 May 1];3:694–704. 10.1038/nrn918 10.1038/nrn918 12209118

[6] Mattson M, Kater S. Calcium regulation of neurite elongation and growth cone motility. J Neurosci [Internet]. 1987 [cited 2021 Jul 2];7:4034–43. https://www.jneurosci.org/lookup/doi/10.1523/JNEUROSCI.07-12-04034.1987 10.1523/JNEUROSCI.07-12-04034.1987 PMC6569087 3121806

[7] Kocsis JD, Rand MN, Lankford KL, Waxman SG. Intracellular calcium mobilization and neurite outgrowth in mammalian neurons. J Neurobiol [Internet]. 1994 [cited 2021 Jul 2];25:252–64. 10.1002/neu.480250306 10.1002/neu.480250306 8195789

[8] Cambray-Deakin MA, Burgoyne RD. Intracellular Ca2 + and N-methyl-D-aspartate-stimulated neuritogenesis in rat cerebellar granule cell cultures. Brain Res Dev Brain Res. 1992;66:25–32. 1600630 10.1016/0165-3806(92)90136-K

[9] Turlova E, Bae CYJ, Deurloo M, Chen W, Barszczyk A, Horgen FD et al. TRPM7 Regulates Axonal Outgrowth and Maturation of Primary Hippocampal Neurons. Mol Neurobiol [Internet]. 2016 [cited 2019 May 1];53:595–610. http://link.springer.com/10.1007/s12035-014-9032-y 10.1007/s12035-014-9032-y PMC4820394 25502295

[10] Launay P, Fleig A, Perraud A-L, Scharenberg AM, Penner R, Kinet J-P. TRPM4 Is a Ca2+-Activated Nonselective Cation Channel Mediating Cell Membrane Depolarization. Cell [Internet]. 2002 [cited 2018 Jul 18];109:397–407. https://www.cell.com/cell/abstract/S0092-8674(02)00719-5 10.1016/s0092-8674(02)00719-5 12015988

[11] Li K, Shi Y, Gonye EC, Bayliss DA. TRPM4 contributes to subthreshold membrane potential oscillations in multiple mouse pacemaker neurons. eNeuro. 2021;8:ENEURO0212–212021. 10.1523/ENEURO.0212-21.2021 PMC8607911 34732535

[12] Cáceres M, Ortiz L, Recabarren T, Romero A, Colombo A, Leiva-Salcedo E et al. TRPM4 Is a Novel Component of the Adhesome Required for Focal Adhesion Disassembly, Migration and Contractility. PLoS ONE [Internet]. 2015;10:e0130540. http://www.ncbi.nlm.nih.gov/pubmed/26110647 10.1371/journal.pone.0130540 PMC4482413 26110647

[13] Blanco C, Morales D, Mogollones I, Vergara-Jaque A, Vargas C, Álvarez A, et al. EB1- and EB2-dependent anterograde trafficking of TRPM4 regulates focal adhesion turnover and cell invasion. FASEB J. 2019;33:9434–52. 31112396 10.1096/fj.201900136R

[14] Riquelme D, Silva I, Philp AM, Huidobro-Toro JP, Cerda O, Trimmer JS et al. Subcellular Localization and Activity of TRPM4 in Medial Prefrontal Cortex Layer 2/3. Frontiers in Cellular Neuroscience [Internet]. 2018 [cited 2018 Jul 18];12:12. http://journal.frontiersin.org/article/10.3389/fncel.2018.00012/full 10.3389/fncel.2018.00012 PMC5797675 29440991

[15] Dotti CG, Sullivan CA, Banker GA. The establishment of polarity by hippocampal neurons in culture. J Neurosci [Internet]. 1988 [cited 2024 Jun 3];8:1454–68. https://www.jneurosci.org/content/8/4/1454 10.1523/JNEUROSCI.08-04-01454.1988 PMC6569279 3282038

[16] Tang F, Dent EW, Kalil K. Spontaneous calcium transients in developing cortical neurons regulate axon outgrowth. J Neurosci. 2003;23:927–36. 12574421 10.1523/JNEUROSCI.23-03-00927.2003 PMC6741922

