# Supplementary material for: TRPM4 inhibition slows neuritogenesis progression of cortical neurons
Source: Mol Brain. 2024 Sep 12;17:66. doi: 10.1186/s13041-024-01140-3 (PMC11391768; doi:10.1186/s13041-024-01140-3)
Supplement: Supplementary file 1 — Supplementary Material 1: Supplementary Methods [file 13041_2024_1140_MOESM1_ESM.docx]

# SUPPLEMENTARY MATERIAL

# METHODS

## Animals

All experiments were conducted following the animal protocols approved by the Ethical committee of the Universidad de Santiago de Chile (N° 426/2019) following the rules and guidelines from the National Agency of Research and Development (ANID). Briefly, C57BL/6J female mice were housed in a temperature and humidity-controlled facility with a 12/12 h light/dark cycle with water and food *ad libitum*.

## Cortical neuron cultures

Primary cortical neurons were prepared from E18 C57BL/6J mice embryos. The cortices were dissected in Hank's Balanced Salt Solution (HBSS) plus 5mM MgCl_2_, pH 7.4; the meninges were removed, and the cortices were minced with tweezers and transfer to a 15 mL tube, washed two times with HBSS and digested with 0.25% trypsin plus 0.03 mg/mL DNase I for 8 min at 37°C. The trypsin solution was washed 3 times plating media, and the tissue was triturated 8 times using a fire polished Pasteur pipette, after decanting the tissue, the supernatant was passed through a 40 µm cell strainer and the cells were counted using a hemocytometer. The neurons were plated in plating media (MEM media supplemented with 20% horse serum [1], 0.1% glucose, 0.5 mM sodium pyruvate, 10 mM HEPES and 100 I.U./mL penicillin-streptomycin) at a density of 50.000 cells per well on 12 mm coverslips pre-coated with 30 µg/mL poly-d lysine and 2 µg/mL laminin until 24 h.

## Neuronal transfection

Neurons were transfected after plating with the shRNA for TRPM4 (shTRPM4) or the scramble sequence (kindly provided by Dr. DJ Linden, John Hopkins University [2]) using Lipofectamine 2000 (Invitrogen/Thermo Fisher). Briefly, 1 µg DNA was mixed with 2.5 µL lipofectamine 2000 in Opti-MEM I and incubated 20 min at room temperature (RT) and then added to the neurons with plating media and incubated for 3 h at 37°C with 5% CO_2_. Next, transfection media was removed and replaced with Neurobasal medium supplemented with 2% B27, 1% GlutaMAX-I and 100 IU / mL penicillin-streptomycin, and neurons were maintained at 37°C in 5% CO_2_ until DIV2.

## Cell treatments

9-Phenanthrol and CBA was prepared according to the manufacturer’s instructions and kept as frozen stock solutions and was added to the plating media at 10 µM or 30 µM after plating until 24 h. In the case of CBA, it was acutely perfused during the Ca^2+^ imaging experiments.

## Immunofluorescence

Neurons were fixed in 4% w/v formaldehyde dissolved in Phosphate-Buffered Saline (PBS) pH 7.4 for 10 min at RT and then washed 3 times in PBS. After washing the cells were permeabilized for 5 min with 0.25% Triton X-100 and blocked 1 h in 10% goat serum. Neurons were incubated with the primary antibodies diluted in 10% goat serum: β tubulin III (1:300;) and TRPM4 (1:100; Origene) overnight at 4 °C. Coverslips were washed 3 times in PBS and then incubated with the appropriate secondary antibodies: Goat anti Mouse IgG1Alexa Fluor 488 or Donkey anti Rabbit IgG Alexa Fluor 488, for 1.5 h at RT and then washed 3 times in PBS. Phalloidin (1:1000) was incubated for 30 min at RT. Coverslips were mounted in Prolong Gold.

## Cell viability

Neurons treated for 1h, 3h or 24 h with 10 µM 9-Phenanthrol were incubated with 0.4% of trypan blue for 5 min and coverslips were observed under an optical microscope. Stained and unstained cells were counted in ten random fields for coverslip. For floating cells, the neurons were collected from the supernatant and then centrifuged 5 min at 500g, then supernatant were discarded, and neurons were incubated with 0.4% of trypan blue and the neurons were counted in a Neubauer chamber. Data were expressed as the percentage of dead cells.

## Ca^2+^_i_ imaging in primary neurons

Cortical neurons in DIV 0 (24h) were incubated with a Krebs buffer (in mM: 140 NaCl, 5 KCl, 2.5 CaCl_2_, 1 MgCl_2_, 10 HEPES, 10 glucose, pH 7.4) containing 3 µM Fluo-4AM for 30 min at RT, then neurons were washed for 30 min with a Krebs buffer at RT. Then, neurons were placed in a Nikon Ti2 microscope, and neurons were imaged using micromanager 2.0 [3] and a Lambda DG4 controlled with an Arduino Uno interface and an ORCA-ER CCD camera. Images were acquired at 10 Hz and fluorescence was measured as the fluorescence intensity over the initial fluorescence (F/F_0_).

## Images acquisition and analysis

Images were acquired in an epifluorescence microscope (Nikon Eclipse) with a 40X objective or in a laser scanning confocal microscope (Zeiss LSM 800) with appropriate excitation and emission filters, 1 AU pinhole, and 40x (1.4 NA) oil immersion objective; laser power and gain settings was adjusted to prevent signal saturation.

Image analysis was performed in ImageJ. Stages of neuronal development were analyzed using the cell counter plugin. Neurons were classified according to the morphological stages previously described by Dotti [4]. Neurite number and length were determined using the NeuronJ plugin.

Transfection efficiency was measured in ImageJ. We defined a somatic ROI in neurons expressing GFP (shRNA for TRPM4) and we transferred this ROI to the image immunostained with TRPM4 antibody. In this image we measured the signal intensity and area. Data were normalized as a percentage of the signal intensity of non-transfected neurons.

## Statistical Analysis

Neuronal stages percentages were analyzed using Two-way ANOVA followed by Šídák's multiple comparisons test. For neurite number, length, transfection efficiency and Ca^2+^ imaging, data normality was determined using the Shapiro-Wilk test. Parametric data was analyzed using t-test and non-parametric data was analyzed using Mann Whitney test.

**REFERENCES**

1. Fedoroff S, Hall C. Effect of horse serum on neural cell differentiation in tissue culture. In Vitro. 1979;15:641–8.

2. Kim YS, Kang E, Makino Y, Park S, Shin JH, Song H, et al. Characterizing the conductance underlying depolarization-induced slow current in cerebellar Purkinje cells. J Neurophysiol. 2013;109:1174–81.

3. Edelstein AD, Tsuchida MA, Amodaj N, Pinkard H, Vale RD, Stuurman N. Advanced methods of microscope control using μManager software. J Biol Methods. 2014;1:e10.

4. Dotti CG, Sullivan CA, Banker GA. The establishment of polarity by hippocampal neurons in culture. J Neurosci . 1988;8:1454–68.
